# Non-invasive ultrasonic neuromodulation of neuronal excitability for treatment of epilepsy

**DOI:** 10.7150/thno.40520

**Published:** 2020-04-12

**Authors:** Zhengrong Lin, Long Meng, Junjie Zou, Wei Zhou, Xiaowei Huang, Shan Xue, Tianyuan Bian, Tifei Yuan, Lili Niu, Yanwu Guo, Hairong Zheng

**Affiliations:** 1 Institute of Biomedical and Health Engineering, Shenzhen Institutes of Advanced Technology, Chinese Academy of Sciences, Shenzhen, China, 518055.; 2 CAS Key Laboratory of Health Informatics, Shenzhen Institutes of Advanced Technology, Shenzhen, China, 518055.; 3 The National Key Clinic Specialty; The Engineering Technology Research Center of Education Ministry of China; Guangdong Provincial Key Laboratory on Brain Function Repair and Regeneration; Department of Neurosurgery, Zhujiang Hospital, Southern Medical University, Guangzhou, China, 510282.; 4 Shanghai Mental Health Center, Shanghai Jiaotong University School of Medicine, Shanghai, China, 200030.; 5 Guangdong-Hong Kong-Macao Greater Bay Area Center for Brain Science and Brain-Inspired Intelligence, Guangzhou, China, 510515

**Keywords:** pulsed ultrasound treatment, epilepsy, electrophysiological activities

## Abstract

Non-invasive low-intensity pulsed ultrasound has been employed for direct neuro-modulation. However, its range and effectiveness for different neurological disorders have not been fully elucidated.

**Methods:** We used multiple approaches of electrophysiology, immunohistochemistry, and behavioral tests as potential epilepsy treatments in non-human primate model of epilepsy and human epileptic tissues. Low-intensity pulsed ultrasound with a frequency of 750 kHz and acoustic pressure of 0.35 MPa (the spatial peak pulse average intensity, I_SPPA_ = 2.02 W/cm^2^) were delivered to the epileptogenic foci in five penicillin-induced epileptic monkey models. An ultrasound neuro-modulation system with a frequency of 28 MHz and acoustic pressure of 0.13 MPa (I_SPPA_ = 465 mW/cm^2^) compatible with patch-clamp systems was used to stimulate the brain slices prepared from fifteen patients with epilepsy.

**Results:** After 30 min of low-intensity pulsed ultrasound treatment, total seizure count for 16 hours (sham group: 107.7 ± 1.2, ultrasound group: 66.0 ± 7.9, P < 0.01) and seizure frequency per hour (sham group: 15.6 ± 1.2, ultrasound group: 9.6 ± 1.5, P < 0.05) were significantly reduced. The therapeutic efficacy and underlying potential mechanism of low-intensity pulsed ultrasound treatment were studied in biopsy specimens from epileptic patients *in vitro*. Ultrasound stimulation could inhibit epileptiform activities with an efficiency exceeding 65%, potentially due to adjusting the balance of excitatory-inhibitory (E/I) synaptic inputs by the increased activity of local inhibitory neurons.

**Conclusion:** Herein, we demonstrated for the first time that low-intensity pulsed ultrasound improves electrophysiological activities and behavioral outcomes in a non-human primate model of epilepsy and suppresses epileptiform activities of neurons from human epileptic slices. The study provides evidence for the potential clinical use of non-invasive low-intensity pulsed ultrasound stimulation for epilepsy treatment.

## Introduction

Epilepsy is one of the most prevalent neurological disorders characterized by recurrent seizures resulting from excessive excitation or inadequate inhibition of neurons 1-5. During the seizures, abnormal synchronized activities in the epileptic focus may spread to other brain regions, causing behavioral disorders 6-8. Neuro-modulation techniques have recently been employed to modulate aberrant neuronal activity and decrease the frequency and/or duration of seizures 9. For instance, vagus nerve stimulation (VNS) is an established and safe procedure to suppress seizure activities by delivering electrical impulses to the brain 10-12. Also, deep brain stimulation (DBS) reduces seizure frequency and severity in both animal and human studies with implantation of one or more electrodes in specific brain regions 13, 14. Optogenetics, the use of light to modulate neural circuits via viral transduction of protein channels, has emerged as a potential method for treating epilepsy. Combined with a closed-loop seizure detection system, optogenetics has been shown to control spontaneous seizures in animal models of epilepsy 7, 15. Besides, transcranial direct current stimulation (tDCS) and transcranial magnetic stimulation (TMS) are non-invasive methods that modulate regional cortical excitability through electric current, and have been shown to effectively decrease epileptic seizures and suppress epileptiform activities 16-18. However, each technique has certain limitations, such as lacking spatial specificity/depth (TMS and tDCS), or containing invasive procedures (DBS and VNS).

Non-invasive low-intensity pulsed ultrasound with high spatial specificity and penetration depth has emerged as a novel neuromodulation technique 19. The ultrasound waves can penetrate the intact skull to specific brain regions, causing modulatory effects of neuronal activity or behavioral outcome 20-29. Pioneering studies in monkeys have provided evidence that low-intensity pulsed ultrasound can be used to modify perception and behavior 22, 26. Also, human studies have demonstrated that low-intensity pulsed ultrasound can non-invasively modulate the function of the primary somatosensory cortex and cause significant changes in electroencephalograph responses by enhancing the discrimination abilities 23, 30. Although recent studies have shown that ultrasound stimulation can suppress epileptic seizures in animal models 28, 31, the therapeutic potential of low-intensity pulsed ultrasound for non-human primate epileptic model or human epileptic tissues remains to be elucidated.

We aimed to investigate whether low-intensity pulsed ultrasound was capable of modulating epilepsy. First, the effectiveness of ultrasound stimulation was identified by a penicillin-induced epilepsy model in non-human primates *in vivo*. Subsequently, brain tissues from patients with temporal lobe epilepsy (TLE) undergoing surgery were tested by miniaturized ultrasound stimulation systems compatible with patch-clamp technique *in vitro*. Our results showed that ultrasound stimulation exerted an inhibitory influence on epileptiform discharges and improved behavioral seizures in a non-human primate epileptic model. Furthermore, ultrasound stimulation could potentially modulate neuronal excitability to inhibit epileptiform activities in human epileptic tissues. The results from epileptic monkey models *in vivo* and human epileptic tissues *in vitro* suggest that low-intensity pulsed ultrasound could suppress epileptiform activities and may provide a potential clinical treatment for epilepsy.

## Materials and Methods

### Ultrasound stimulation in monkeys

The study was approved by the Ethics Committee of the Center of Guangdong Landao Biotechnology in Guangzhou, China (LDACU20170306-01) and was performed in accordance with recommendations from the Guidelines for the Use and Care of Experimental Animals. Every effort was made to minimize suffering. Five monkeys (Macaca fascicularis, labeled from 1 to 5, 4-7 years old, 5.4-5.9 kg) were provided by the Center of Guangdong Landao Biotechnology in Guangzhou, China. They were individually housed in a temperature (24 ± 1°C) and humidity (50 ± 5%) controlled facility with a 12 h light dark cycle (lights on 8:00 a.m.). Each monkey had free access to standard primate chow and water.

**Figure 1:**
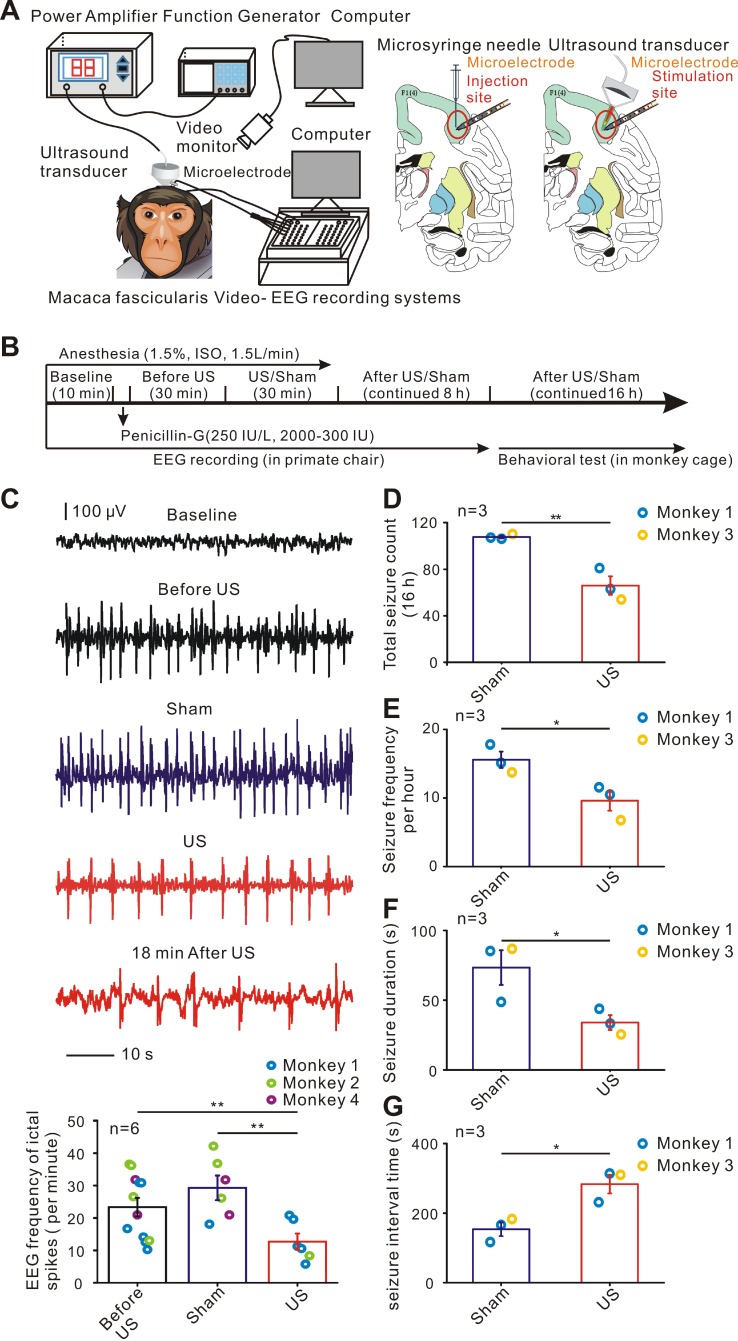
Ultrasound stimulation improves electrophysiological activities and behavioral outcomes in penicillin-induced epileptic monkey models. (A) Schematic illustration depicting the system used for stimulating the monkey. A single-element focused ultrasound transducer with fundamental frequency of 750 kHz, acoustic pressure of 0.35 MPa (I_SPPA_= 2.02 W/cm^2^), TBD of 300 μs, PRF of 1000 Hz, SD of 200 ms, and ISI of 5 s was placed on the site of penicillin injection to deliver ultrasound energy to the epileptogenic foci (The right frontal lobe). A depth-microelectrode was placed to record the electrophysiological activities. (B) Flowchart of the experimental procedure. (C) Representative EEG traces in the baseline, Before US, US, Sham and After US indicate that a 30-min US stimulation decreased ictal spike activities in penicillin-induced epileptic monkey models. (D-G) Bar charts of the data measuring different behavioral seizure parameters in the US and Sham groups. D, total seizure count (16 hours); E, seizure frequency per hour; F, seizure duration; G, seizure interval time. The results showed that ultrasound stimulation enabled to improve the behavioral outcomes in penicillin-induced epileptic monkey models. * *P* < 0.05, ** *P* < 0.01.

The effects of ultrasound stimulation on epileptic monkey models were assessed by electrophysiological recording and behavioral outcome analysis. A single-element focused ultrasound transducer (H116, Sonic Concept) was placed on the site of penicillin injection to deliver ultrasound energy to the epileptogenic foci (Figure 1A; see also in Supplementary Figure S1). We performed a total of 12 electrophysiological trials in three monkeys, six sham trials without ultrasound stimulation, six with 30 min ultrasound stimulation, and six behavioral monitoring in two monkeys. Anesthesia was induced by injection of ketamine (10 mg/kg, i.m.) and atropine (0.05 mg/kg, i.m.) for 1.5 hours. The heads of the monkeys were fixed, and surgery was performed using a stereotaxic apparatus (68901, RWD) for nonhuman primates. All hair of the monkeys was shaved, and the skin was fully disinfected and separated to expose the skull. The right frontal lobe was targeted according to the Macaca fascicularis Brain in Stereotaxic Coordinates 32, 33, with the stereotactic coordinates 30 mm posterior to the bregma, 15 mm lateral to the midline, and 3 mm from the dura (Figure 1A). A section of the skull, called a bone flap about 30 mm * 15 mm, was removed to deliver drugs locally. The monkeys were given sufficient nutrition after surgery and antibiotics were applied for 7 days.

Experiments on the monkeys were started after a 14-day postoperative recovery period. Penicillin was diluted to 250 IU/uL, and 2000 to 3000 IU was applied to the brain surface using an injection cannula with a microsyringe (50 μl, 1705RN, Hamilton) at 1μl/min. Focal seizures were induced by penicillin injection in the right frontal lobe (3mm depth) lasting less than 48 hours. After penicillin injection, the monkeys were monitored for epileptiform activities with video-EEG for 8 hours paying attention to containment and comfort of the animal in a restrained position 34, 35. Subsequently, we brought back the monkeys in the cage to monitor behavioral seizures using video recording for 16 hours. Behavioral seizure evaluation index included seizure count in 16 hours, frequency per hour, and duration and seizure interval time which was calculated by the average time between two seizures. A single-element ultrasound transducer (H116, Sonic Concept) with a fundamental frequency of 750 kHz, TBD of 300 μs, PRF of 1000 Hz, sonication duration (SD) of 200 ms, and inter-stimulation interval (ISI) of 5 seconds was used for stimulation of the epileptic focus. The monkeys were treated for 30 min with ultrasound treatment (Figure 1B). In the sham stimulation group, the ultrasound transducer was fixed to the monkeys similar to the stimulated group for 30 minutes without ultrasound signal output. The ultrasound transducer was fixed on the operating arm of the stereotactic device, the probe and the dura mater were kept perpendicular, and the coupling agent was filled between the ultrasonic probe outlet and the membrane meninges. One experiment in monkey 5 was carried out to evaluate the effect of any audible sound produced by ultrasound transducer on epileptiform activities. The ultrasound transducer was placed outside away from the monkey brain and the monkey was monitored for epileptiform activity for 8 h continuously. The transducer was actuated by an electrical signal generated by a function generator (AFG3101, Tektronix) which was concurrently amplified using a power amplifier (2100L, E&I, NY, USA).

After the end of the ultrasound stimulation, we removed the monkeys from the stereo positioner and moved them to the monkey chair, fixing the limbs to the monkey chair with a collar on the neck and the collar connected to the chair. The acoustic intensity (I_SPPA_) was evaluated to be approximately 2.02 W/cm^2^ (the acoustic pressure = 0.35 MPa), as measured with a needle hydrophone (Precision Acoustics, Dorchester, Dorset, UK). Ultrasound pressure mapping was acquired by a commercial finite element method (FEM) software COMSOL Multiphysics and the acoustic field was measured in XY plane by the OptiSon® Ultrasound Beam Analyzer (Onda, USA). The electrophysiological data from monkeys 1, 2, 4, and 5 and behavioral seizure data from monkeys 1, and 3 were further calculated.

### Evaluation of temperature elevation and safety in the monkeys

To evaluate the thermal effect of the ultrasound, we placed a 5mm polyvinyl alcohol on the probe exit plane and recorded the temperature of the ultrasonic probe exit using a thermal infrared imager (R300, NEC Avio, Tokyo, Japan).

One epileptic monkey model after 30 min ultrasound stimulation was sacrificed and histological examinations were performed to evaluate the safety of ultrasound stimulation. Following deep anesthesia, 0.9% NaCl and 4% paraformaldehyde (ph 7.4) solution were immediately perfused through the heart. After fixation, the brain was removed and immersed in 4% paraformaldehyde. The coronal section was taken in the prefrontal cortex area by ultrasound stimulation. Brain sections were then subjected to histological staining and were analyzed using a conventional microscope.

### Evaluation of epilepsy patients

This study was reviewed and approved by the Ethics Committee of the Zhujiang Hospital of Southern Medical University, Guangzhou, China (2016-SJWK-005). Written informed consent and a statement confirming consent to publish were obtained from 19 participants. A total of 15 TLE patients and 4 glioma patients without epilepsy were included in the preliminary study. The details of these patients are summarized in Supplementary Table S1. Based on the systematic assessment, including magnetic resonance imaging, the scalp electroencephalograms (EEGs), semiology, and pathology, the patients were diagnosed with medically intractable focal epilepsy in the temporal lobe (Supplementary Figure S4). Consistent with previous studies, the histopathological findings showed the key features of TLE, including neuronal loss, the layer dispersion, cellular morphology changes, reactive gliosis, and focal cortical dysplasia 36-38. Moreover, epileptiform discharges in the TLE patient were observed in inter-ictal scalp EEGs. Hematoxylin and Eosin (HE) staining and Bielschowsky silver staining demonstrated that neuronophagia and microvacuoles occurred in the neurons of epileptic tissues (Supplementary Figure S4D-E).

### Preparation of brain slices

Biopsy specimens removed from 15 TLE patients or 4 glioma patients without epilepsy were rapidly immersed in ice-cold, oxygenated high-sucrose solution (0 - 2°C) containing (in mM): 60 NaCl, 3 KCl, 7 MgCl_2_, 1.25 NaH_2_PO_4_, 25 NaHCO_3_, 10 D-glucose, 115sucrose, and 0.5 CaCl_2_. Coronal slices (300 μm thick) were prepared with a Vibratome (VT-1200 Series, Leica) instrument. The brain slices were equilibrated and incubated in the ACSF containing (in mM): 126 NaCl, 2.5 KCl, 1 MgCl_2_, 1.25 NaH_2_PO_4_, 26 NaHCO_3_, 10 D-glucose, 2 sodium pyruvate, 0.5 L-ascorbic acid, and 2 CaCl_2_ and saturated continuously with 95% O_2_ - 5% CO_2_, pH 7.3-7.4. The osmolality was 290 - 300 mOsm/L and the temperature was kept at 35°C before the slices reached the recording chamber. Ultrasound stimulation was shown to be effective in exciting or reversibly suppressing neuronal activity. For the selection of ultrasonic parameters, four C57BL/6 mice were sacrificed under deep anesthesia with 20% urethane (10 mL kg^-1^) and slices were prepared as described above. All procedures were performed according to the guidelines of The Institutional Review Board at Zhujiang Hospital of Southern Medical University, Guangzhou, China and the Committee for Animal Experimentation at Shenzhen Institutes of Advanced Technology, Chinese Academy of Sciences.

### Application of ultrasound stimulation in epileptic slices

An ultrasound neuro-modulation chip with 28 MHz resonant frequency compatible with the patch-clamp systems due to the small size and transparent character was used for stimulation of slices *in vitro* (Figure S5)^39^-^41^. The chip consisted of interdigital transducers (IDTs) and a recording chamber (a polydimethylsiloxane ring-shaped chamber). The finger-electrodes of IDTs were deposited using micro-electromechanical systems (MEMS) techniques on a piezoelectric 128^o^ Y-rotated, X-propagating lithium niobate (LN, LiNbO_3_) sub-strate (1 mm thick, transparent) with an aluminium layer of 200 nm 42. Pulsed ultrasound waveforms were generated by an arbitrary waveform generator (AFG 3102, Tektronix, Beaverton, Oregon) and amplified by a power amplifier (ZHL-1-2W+, Mini-Circuits, Brooklyn, NY, USA). The displacement of the piezoelectric substrate perpendicular to the surface plane was 20 pm measured by a Laser Doppler Velocimetry (UHF-120 Ultra High-Frequency Vibrometer, Polytec, Germany) and the acoustic pressure equal to 0.13 MPa (the spatial-peak pulse-average intensity (I_SPPA_) was evaluated to be approximately 465 mW/cm^2^. The spatial peak time average intensity (I_SPTA_) was equal to 233 mW/cm^2^ and was calculated by multiplying duty cycle to the I_SPPA_ 43.

### Electrophysiological recording and histology

The brain slices were transferred to the recording chamber in the ultrasound neuro-modulation chip after 1-hour incubation and were perfused with ACSF flowing at a rate of 2 - 3 ml/min and maintained at 30°C by an automatic temperature controller (TC-324C, WARNER) throughout the experiment. Traditional cell-attached and whole-cell recordings were performed to record the spontaneous activity using a patch-clamp system (AXON 1550A and 700B, USA). Records were filtered at 5 kHz and digitized at a sampling rate of 3 kHz. The series resistance was compensated, and leakage and capacitive currents were subtracted on-line. Patch glass microelectrodes were pulled by a micropipette puller (P-97, Sutter Instrument Co., Novato, CA, USA) and the resistance ranged from 5 to 10 MΩ after filling with the internal solution.

Upon the traditional cell-attached recordings, the spontaneous activity was recorded before and after ultrasound stimulation in 60 seconds duration. The internal solution contained the following (in mM): 126 NaCl, 2.5 KCl, 1 MgCl_2_, 1.25 NaH_2_PO_4_, 26 NaHCO_3_, 10 D-glucose, 2 sodium pyruvate, 0.5 L-ascorbic acid, and 2 CaCl_2_. Whole-cell current-clamps were used to record evoked action potentials in response to different injection currents (ranging between -100 and 400pA). The voltage-clamp internal solution contained the following (in mM): 140 K-gluconate, 4.5 MgCl_2_, 5 EGTA, 4 Mg-ATP, 0.3 GTP, 4.4 phosphocreatine disodium salt hydrate, and 9 HEPES. Further identification of neuronal morphology was carried out by intracellular injection of 0.25% biocytin (Sigma, USA). Whole-cell recordings in the voltage-clamp mode were used to record isolated excitatory and inhibitory postsynaptic currents (EPSCs and IPSCs) at holding potentials of -70, and 0 mV. The current and voltage (I-V) curves showed a linear correlation of postsynaptic currents with the holding potentials. In the -70-mV holding membrane potential, which was close to the reversal potentials for GABA_A_ receptor-mediated Cl^-^ currents, only the EPSCs could be recorded. In the 0-mV holding membrane potential, which was close to the reversal potentials for glutamatergic currents, only the IPSCs could be recorded. To further verify the recording currents, we found that the EPSCs could be fully blocked by applying DNQX (6.7-dinitroquinoxaline-2.3-dione) and AP-5 (DL-2-amino-5-phosphonopentanoic acid). The recording of IPSCs could also be blocked by strychnine and bicuculline. The voltage-clamp internal solution contained the following (in mM): 125 cesium gluconate, 5 TEA-Cl, 4 MgATP, 0.3 GTP, 10 phosphocreatine, 10 HEPES, 10 EGTA, 2 CsCl, 1.5 QX-314, and pH 7.3. Using the same method, the EPSCs and IPSCs were separated in the 'epileptic neurons ' of TLE slices by holding at different membrane potentials.

Histological experiments were carried out to identify the neuronal morphology. Human brain slices of patients were immersed with 4 % paraformaldehyde (for neuronal morphology, 15 min after patch-clamp recording). Brain sections were stained for neuronal morphology (biocytin, Sigma) and pathological assessments (HE staining and Bielschowsky silver staining). The total biopsy specimens for slice preparations were harvested from 15 patients with temporal lobe epilepsy and 4 glioma patients without epilepsy. Biopsy specimens removed from 6 TLE patients and 2 gliomas patients were used for slice preparations for the traditional cell-attached recordings. Biopsy specimens removed from 4 TLE patients and other 2 gliomas were used to prepare slices for whole-cell recordings in the voltage-clamp mode to isolate excitatory and inhibitory postsynaptic currents. Biopsy specimens removed from 2 TLE patients were used for slices preparations for whole-cell recordings in the current-clamp mode to investigate the neuronal excitability in different type of neurons. Some leftover tissues were used for histological experiments. Biopsy specimens removed from 3 TLE patients were used to prepare slices for patch-clamp recording to evaluate temperature elevation produced by ultrasound transducer on epileptiform activities.

### Statistical analysis

All statistical procedures were performed using SPSS (13.0) statistical software package. Values were expressed as means ± standard error of the mean (SEM). The effects of the ultrasound stimulation on spike frequency and amplitude were statistically evaluated by the Pearson correlation coefficient, Student's paired *t*-test, and independent samples *t-*test. One-way ANOVA followed by LSD was used for differences between groups. Statistical significance was defined as a value of *P* < 0.05.

## Results and Discussion

### Ultrasound stimulation reduces epileptiform activities and behavioral seizures in epileptic monkeys

Although non-invasive low-intensity pulsed ultrasound is often used for modulation of different neurological disorders as well as for direct neuromodulation, its range and effectiveness for epilepsy in non-human primate models have not been elucidated. Therefore, we first tested whether ultrasound stimulation could influence epileptiform activities in epileptic monkeys (Figure 1A-B, Supplementary Figure S1). Ultrasound stimulation for 30 minutes effectively suppressed electrographic frequency of ictal spikes per minute in all monkeys tested (Sham: 29.3 ± 3.8; US: 12.62 ± 3.9. n = 6 experiments, *P* < 0.01, independent samples *t-*test, Figure 1C). For behavioral testing, a total of six experiments were performed in two monkeys including three sham experiments without ultrasound stimulation and three with ultrasound stimulation. The mean values of the total seizure counts in 16 hours were significantly reduced (107.7 ± 1.2 in the sham group and 66.0 ± 7.9 in the ultrasound group, n = 2 animals, *P* < 0.01, independent samples *t*-test, Figure 1D) upon ultrasound stimulation. Independent samples *t*-test revealed that the monkeys in the ultrasound stimulation group had lower seizure frequency per hour, lower seizure duration, and longer seizure interval (all *P* < 0.05, Figure 1E-G). These results showed that ultrasound stimulation could effectively suppress epileptiform activities and improve the behavioral outcome in epileptic monkey models. To evaluate the safety of 30 minutes ultrasound stimulation in epileptic monkeys, temperature monitoring and Hematoxylin and Eosin (HE) staining were performed for evidence of tissue damage. Relatively small temperature elevation (0.69 ± 0.044°C) and intact cortex structure and neurons indicated that 30 min ultrasound stimulation in this study was safe for the treatment in epileptic monkey models (Supplementary Figure S2).

Previous studies have suggested two possible explanations for ultrasound neuro-modulation: ultrasound directly activates a localized area or indirectly impacts auditory pathways which further propagate to other cortical networks 44-46. The effect of ultrasound-induced auditory indirect activation is mainly related to acoustic parameters, especially pulse repetition frequencies (PRF). Although our experiment in epileptic monkey models employed ultrasound PRF of 1000 Hz within the hearing range of primates, another experiment was carried out to evaluate the effect of any audible sound produced by ultrasound transducer on epileptiform activities. The ultrasound transducer was placed outside away from the monkey brain and the monkey was monitored for epileptiform activity for 8 h continuously (Supplementary Figure S3A). The result showed that ultrasound had no significant effect on the epileptiform activities of epileptic monkey (Supplementary Figure S3B). Further investigation is needed to clarify whether or not the inhibition of epileptiform activities or behavioral seizures in monkey models is induced by the possible effect of ultrasound-induced auditory indirect activation. Besides, ultrasound exposure promoted drug accumulation in cells through downregulation of P-glycoprotein. Thus, modulation of p-glycoprotein expression might be part of the ultrasound-induced effects 47.

### Ultrasound stimulation directly inhibits the epileptiform discharges in neurons from TLE patients

**Figure 2:**
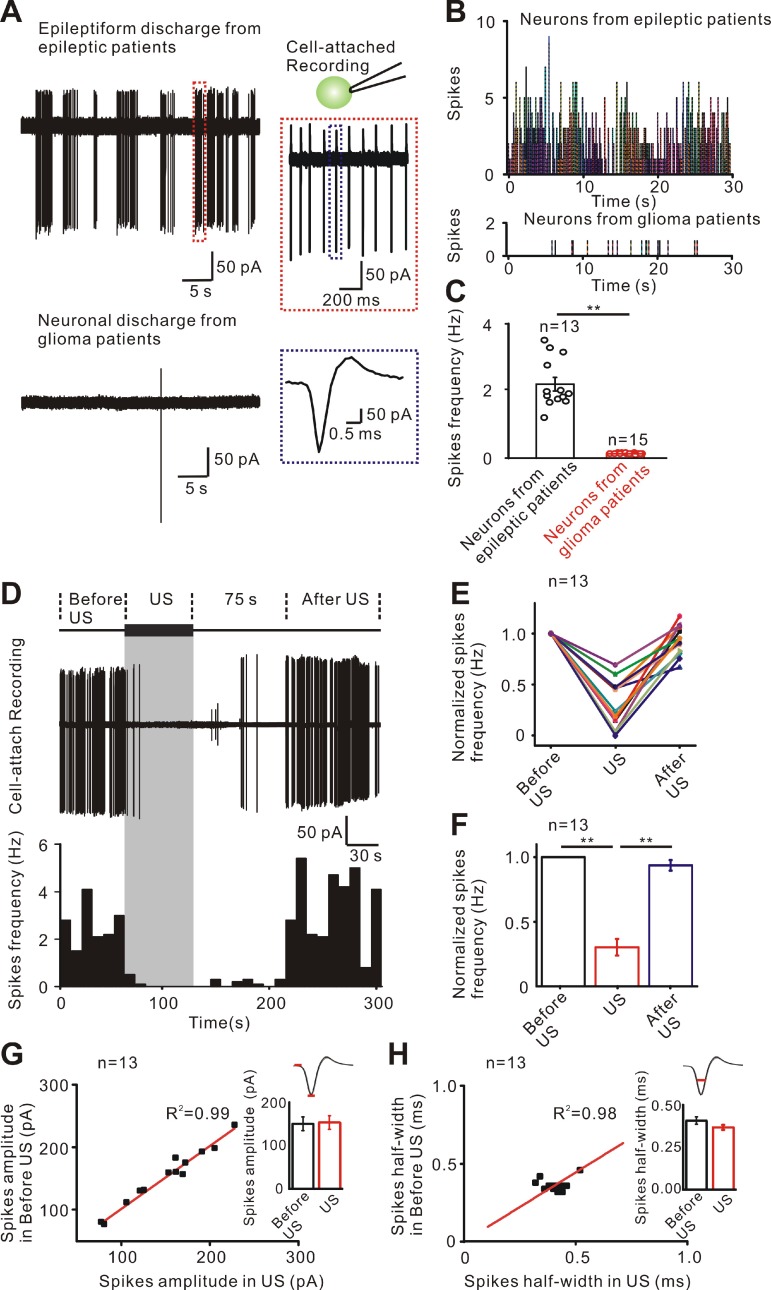
Inhibitory effect of ultrasound stimulation on epileptiform discharges of TLE patients. (A) Cell-attached recording of neurons from epileptic patients has epileptiform discharges consisting of few to dozens of spikes while neurons from glioma patients without epilepsy have few discharges. (B) PSTH (pre-stimulation spikes time histogram) of 13 'epileptic neurons' and 15 normal neurons showing the epileptiform discharges in the 'epileptic neurons' of TLE patients compared with the few discharges in glioma patients. (C) Comparison of the frequency of spikes showing that the firing frequency of neurons from epileptic patients was significantly higher than that of neurons from glioma patients. (D) Representative traces of epileptiform discharge recorded (middle) in different recording phases (upper, 60 seconds before ultrasound stimulation, 60 seconds ultrasound stimulation and after ultrasound stimulation). Spike frequency of epileptiform discharges was significantly decreased during ultrasound stimulation (lower). The inhibitory effect lasted 75 seconds and rapidly resumed after ultrasound stimulation. (E) Thirteen neurons recorded from TLE patient's slices showing an effective decrease in the frequency of epileptiform discharges during ultrasound stimulation. The frequency of epileptiform discharges resumed after ultrasound stimulation. (F) Ultrasound stimulation significantly decreased the normalized spikes frequency of epileptiform discharges. (G) No significant change was observed before US and during US in the amplitude of the spikes. (H) No significant change was observed before US and US in the half-width of spikes. Sixty seconds pulsed ultrasound waveforms with a fundamental frequency of 28 MHz, acoustic pressure of 0.13 MPa (I_SPPA_= 465 mW/cm^2^), TBD of 5 ms and PRF of 100 Hz were used to stimulate the human epileptic slices.

To investigate whether ultrasound stimulation could suppress epileptiform activities in human epileptic slices and uncover the underlying mechanisms, an experimental system consisting of an ultrasound neuro-modulation chip and patch-clamp systems was used for stimulation of brain slices *in vitro* (Supplementary Figure S5). Using cell-attached recording, we first detected the epileptiform discharges in neurons from TLE patients while there were few spontaneous discharges in neurons from glioma patients (Figure 2A-C). The neurons from TLE patients produced a 0.3 - 0.5 Hz epileptiform discharge with few to dozens of spikes (n = 13), while the neurons in glioma patients did not show such epileptiform discharges (n = 15). The pattern of epileptiform discharges in neurons from human epileptic tissue *in vitro* and those seen in neurons recorded in both acute and chronic epileptogenic foci *in vivo* was similar^48^, ^49^.

Previous studies have indicated that acoustic parameters are associated with the bimodal modulatory effects of ultrasound on neuronal activity 50-52. We used mice brain slices to test the excitatory or inhibitory effect of ultrasound stimulation with different acoustic parameters (Supplementary Figure S6). Sixty second low-intensity pulsed ultrasound waveforms with frequency of 28 MHz, I_SPPA_ of 465 mW/cm^2^ tone burst duration (TBD) of 5 ms and pulse repetition frequency (PRF) of 100 Hz indicated inhibitory effect on neuronal excitability in mice cortical slices and were, therefore used to modulate the human epileptic slices. Figure 2D shows that the epileptiform discharges of neurons could be effectively inhibited during ultrasound stimulation. The inhibitory effect lasted about 75 seconds, and then rapidly resumed after ultrasound stimulation. The histogram of spikes frequency displays the inhibitory process of ultrasound stimulation. Recording from a population of 13 neurons from TLE patients showed that the relative frequency of epileptiform discharges rapidly decreased several-fold (Figure 2E) and the inhibitory effect lasted 63 ± 4 seconds after ultrasound stimulation. As displayed in Figure 2F, ultrasound stimulation significantly decreased the relative frequency of spikes compared to before ultrasound stimulation (during US: 0.303 ± 0.064 Hz; after US: 0.936 ± 0.041 Hz. *P* < 0.01, one-way ANOVA followed by LSD). Furthermore, analysis of individual spike waveform revealed that both the amplitude and half-width were unchanged before and during ultrasound treatment (150.16 ± 12.77 pA to 152.45 ± 12.27 pA, *P* = 0.18 for mean amplitude of spikes; 0.41 ± 0.015 ms to 0.36 ± 0.011 ms, *P* = 0.13 for mean half-width of spikes, Figure 2G-H). Previous results indicated that disruptions of the synaptic inputs could rapidly produce epileptiform activities and seizures within minutes 53, 54. To quickly re-stabilize neural circuits before the onset of pathological activities, an effective method is required for compensatory adjustments of synaptic inputs. In cultured hippocampal and cortical neurons, restoration of the synaptic inputs occurred over hours to days through homeostatic adjustments 55-58. Ultrasound stimulation used in this study could quickly adjust the synaptic inputs within seconds. Therefore, noninvasive ultrasound stimulation might have significant translational therapeutic potential as an antiepileptic treatment modality.

The ultrasound wave used in this study was generated by an ultrasound neuro-modulation chip (fundamental frequency, 28 MHz). The obvious question then was whether the chip had the same effect on neurons as the traditional ultrasound transducer used in the non-human primate model of epilepsy (fundamental frequency, 750 kHz). Our previous studies have demonstrated that the ultrasound neuro-modulation chip transmitting surface acoustic waves (SAW) could directly modulate the neurons of hippocampal slices or from *C. elegans* 39, 41. When the SAW propagates along the substrate entering the confined fluid in the microcavity, the refraction of SAW occurs at the interface of the fluid and substrate. Part of the SAW energy converts to bulk wave propagating in the fluid, and the rest of acoustic energy lies in the leaky SAW propagating in the substrate 59-61. Furthermore, SAW as a form of ultrasound has similar bio-effects on the cells compared to the longitudinal wave generated by the traditional ultrasound transducer 59, 62. Therefore, due to its compatibility with patch clamp and standard calcium imaging, the ultrasound neuro-modulation chip was first used to study the inhibitory effects of ultrasound stimulation and the underlying mechanism on inhibiting epileptiform discharges.

### Ultrasound stimulation re-adjusts the imbalance of synaptic inputs to inhibit the epileptiform discharges

**Figure 3:**
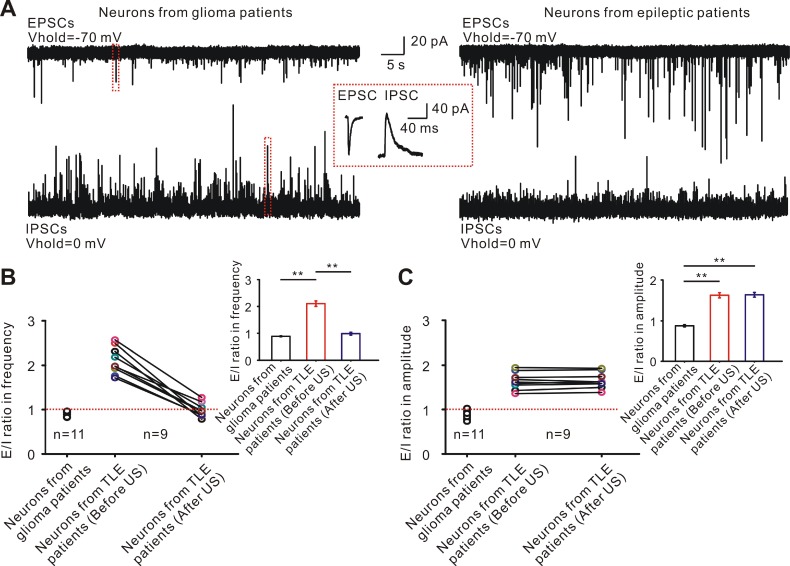
Ultrasound stimulation re-adjusts the imbalance of excitatory and inhibitory inputs in the neurons from TLE epileptic slices. (A) Representative current traces of EPSCs and IPSCs were recorded in the neurons from TLE patients and neurons from glioma patients separated by holding in different membrane potentials. (B-C) Nine neurons from TLE epileptic slices showed an imbalance in E/I of frequency or amplitude compared with the neurons from glioma patients (unpaired *t* test, *P* < 0.01). Ultrasound stimulation re-adjusted the balance of E/I in frequency, but not amplitude (10 neurons from TLE epileptic slicesultrasound stimulation in the E/I of frequency, paired *t* test, *P* < 0.01; 1.64 ± 0.06 to 1.65 ± 0.06 after 60 seconds ultrasound stimulation in the E/I of amplitude, paired *t* test, *P* = 0.37 ).

The interplay of cortical excitation and inhibition is a fundamental feature of cortical function processing 63, 64. The cortical neuronal activity is dynamically adjusted by synaptic inputs, including the excitatory postsynaptic currents (EPSCs) and inhibitory postsynaptic currents (IPSCs), which is often disrupted in the neurons of TLE patients 65-68. To test whether disturbed synaptic inputs in the neurons from TLE patients can be adjusted by ultrasound stimulation, the EPSCs and IPSCs of the neurons from TLE patients (n = 9) and glioma patients (n = 11) were separated by holding the neurons at different membrane potentials. A synaptic input was observed in 11 neurons from 4 glioma patients (Figure 3A left) while both the frequency and amplitude of the EPSCs were higher than those of IPSCs in the neurons from TLE patients (Figure 3A right). Ultrasound stimulation progressively restored the imbalance of E/I frequency from 2.10 ± 0.10 Hz to 0.99 ± 0.05 Hz (*P* < 0.01, paired t test, Figure 3B). However, ultrasound stimulation did not change the E/I of amplitude (Before US: 1.64 ± 0.06, after US: 1.65 ± 0.06, *P* = 0.19, paired t test, Figure 3C).

**Figure 4:**
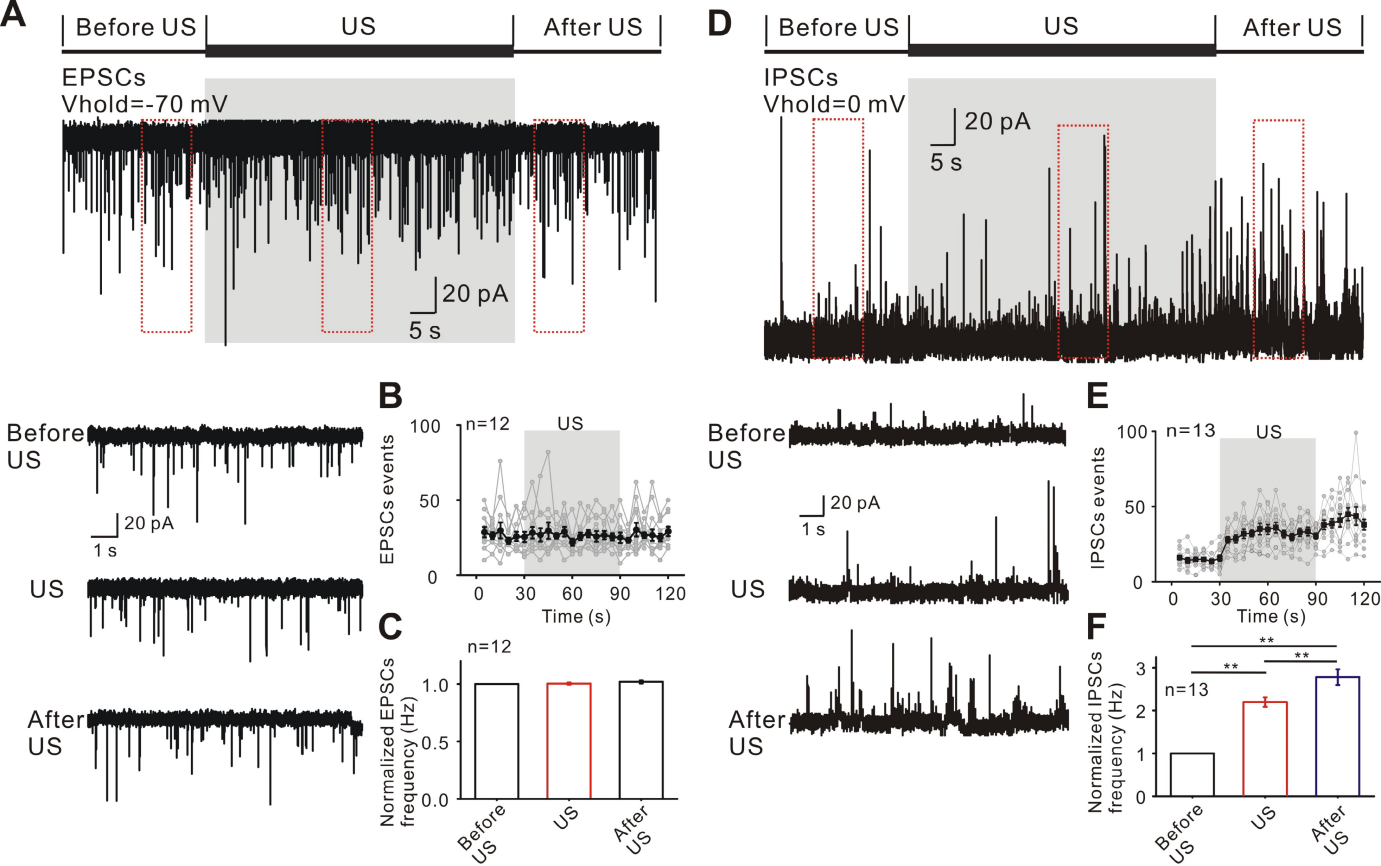
Ultrasound stimulation directly increases the inhibitory synaptic inputs. (A) Representative current traces of spontaneous EPSCs recorded in the neurons from TLE epileptic slices in different recording phases. (B-C) Ultrasound stimulation did not change the events and normalized frequency of EPSCs in neurons. (D) Representative current traces of spontaneous IPSCs recorded from TLE epileptic slices in different recording phases. (E-F) Ultrasound stimulation significantly increased the events and normalized frequency of IPSCs.

The observed inhibitory effect of epileptiform discharges induced by ultrasound stimulation might be result from reduced excitatory inputs, increased inhibitory inputs or modulating both excitatory and inhibitory inputs. To test this hypothesis, the holding membrane potential was set at -70 mV for recording excitatory post-synaptic currents during three phases (Figure 4A). Ultrasound stimulation had no significant effect on the events and relative frequency of EPSCs compared to those before ultrasound (US: 1.024 ± 0.035 Hz; after US: 1.029 ± 0.016 Hz; *P* = 0.49, one-way ANOVA followed by LSD, n = 12, Figure 4B-C). Furthermore, analysis of the EPSCs waveform revealed that the mean of amplitude, 10% - 90% rise time, and half-width were unchanged before ultrasound and in ultrasound stimulated groups (Supplementary Figure S7A).

We further tested the effects of ultrasound stimulation on inhibitory synaptic activities. The holding membrane potential was set at 0 mV for recording inhibitory post-synaptic currents during three phases (Figure 4D). A population of 13 neurons from TLE patients showed a rapid and significant increase in the relative frequency of IPSC events (Figure 4E) compared to before ultrasound (US: 2.467 ± 0.075 Hz; after US: 3.241 ± 0.143 Hz; *P* < 0.01, one-way ANOVA followed by LSD, Figure 4F). As was the case with EPSCs, there was no change in the mean of amplitude, 10%-90% of rise time, and half-width in IPSC waveform before and after the ultrasound stimulation (Supplementary Figure S7B). Pretreatment with SR-95531, a specific GABA receptor antagonist, prevented the effect of ultrasound stimulation on inhibition of epileptiform activities (Supplementary Figure S8). Treatment with the GABA receptor antagonist hardly changed the relative frequency of ultrasound stimulation (during US: 1.02 ± 0.027 Hz; After US: 1.04 ± 0.026 Hz; *P* = 0.29, one-way ANOVA followed by LSD, Supplementary Figure S8A-C) and individual spike waveform (326.18 ± 46.43 pA to 328.54 ± 41.62 pA, *P* = 0.31, Student's paired t-test for mean spikes amplitude; 0.525 ± 0.03 ms to 0.515 ± 0.05ms, *P* = 0.39, Student's paired t-test for mean spikes half-width, Supplementary Figure S8D-E). The results demonstrated that ultrasound stimulation could induce an increase in inhibitory inputs, thus adjusting the synaptic inputs to inhibit the epileptiform discharges.

### Ultrasound stimulation activates the interneurons to increase the inhibitory synaptic inputs

**Figure 5:**
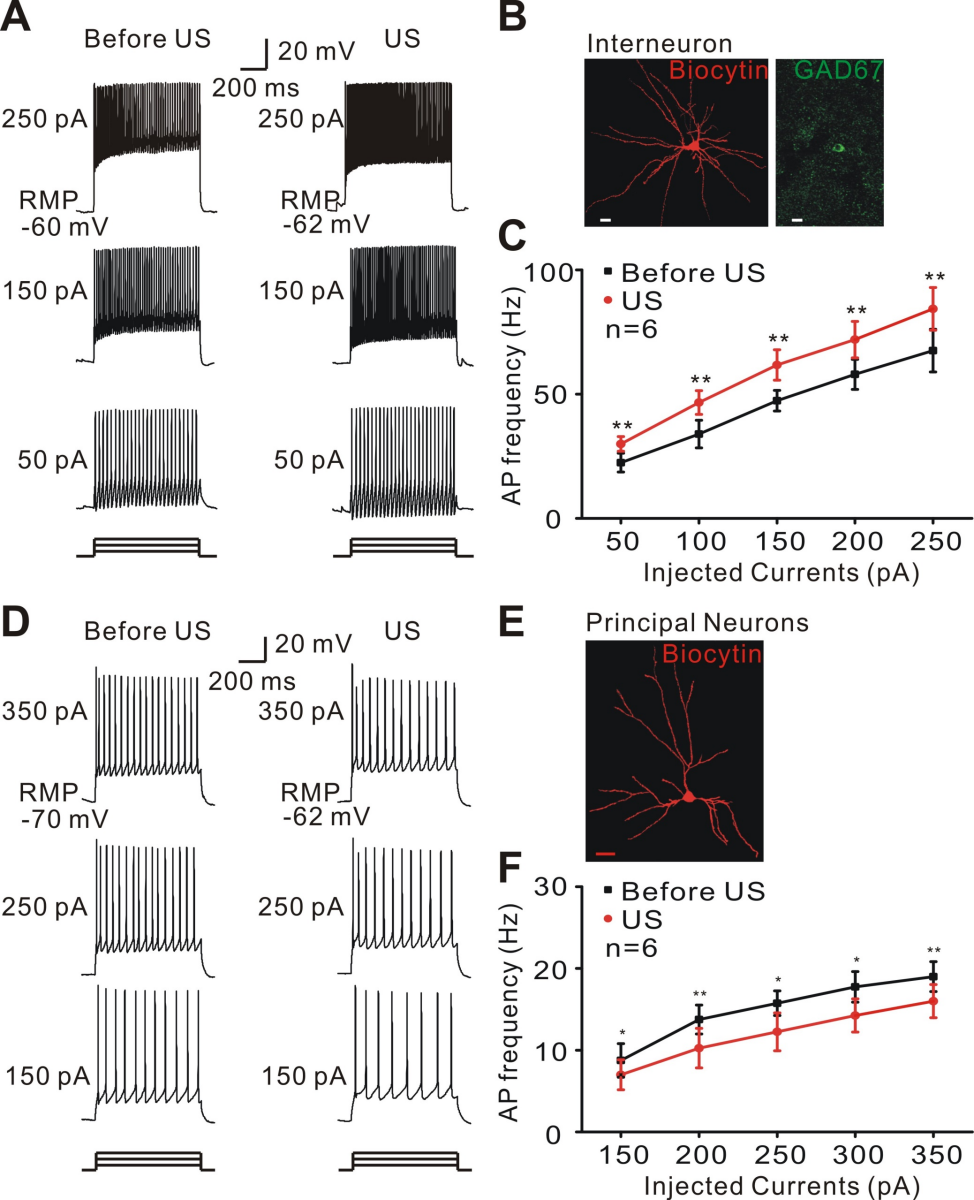
Ultrasound stimulation modulates the neuronal excitability in epileptic slices. (A) Representative voltage traces recorded from interneurons of epileptic slices in response to a sequence of sustained currents injection (50, 150, and 250 pA). During ultrasound stimulation, interneurons could activate more action potentials. (B) Double-staining of biocytin-injected neurons with GAD 67 antibody showing a typical morphology of interneuron. Scale bar = 10 μm. (C) Ultrasound stimulation (red) significantly increased the firing frequency of interneurons compared with Before US (black, Student's paired t-test, ** *P* < 0.01). (D) Representative voltage traces recorded from pyramidal neurons of epileptic slices in response to a sequence of sustained currents injection (150, 250, and 350 pA). Ultrasound stimulation suppressed the firing frequency of pyramidal neurons. (E) Biocytin-injected staining showing a typical morphology of pyramidal neurons. Scale bar = 20 μm. (F) Ultrasound stimulation (red) significantly decreased the firing frequency of pyramidal neurons compared with Before US (black, Student's paired t-test, * *P* < 0.05; ** *P* < 0.01).

We tested the possibility that ultrasound stimulation directly activated the interneurons of epileptic tissues to increase inhibitory inputs. Whole-cell patch-clamp recording of typical interneurons was identified by morphology and firing pattern in response to injected currents and indicated that ultrasound stimulation increased the inter-neuronal excitability of epileptic tissues (Student's paired *t-*test, ** *P* < 0.01, Figure 5A-C). Consistent with these findings, ultrasound stimulation decreased the activity of pyramidal neurons in epileptic slices (Student's paired *t-*test, * *P* < 0.05, ** *P* < 0.01, Figure 5D-F).

Three potential physical mechanisms could contribute to the modulation of neuronal excitability induced by ultrasound stimulation including thermal, mechanical, and cavitation effects 69. Pulsed ultrasound stimulation with 60 second duration could accumulate heat in the recording chamber and the temperature elevation might exert an important influence on the activity of neurons 20, 70, 71. The results showed that the temperature elevation during the perfusion of artificial cerebrospinal fluid (ACSF) during ultrasound stimulation was relatively small (less than 0.64 ± 0.036°C; Supplementary Figure S9). To evaluate the effect of temperature induced by ultrasound, we also stimulated the neurons obtained from human epileptic slices using heated ACSF (2°C temperature elevation) in a water bath. The results showed that 2°C temperature elevation of ACSF perfusion during the recording had no significant effect on the spontaneous activity and evoked firing of recording neurons in TLE slices (Supplementary Figure S10), indicating that temperature elevation of 0.64 ±0.036 °C might not inhibit the epileptiform discharge in human TLE slices. The frequency used in the brain slices (28 MHz) was relatively high. Therefore, we also fabricated a neuro-modulation chip with **a** frequency of 6.57 MHz to investigate the effect of the frequency on the suppression of neuronal activities in pyramidal neurons from human epileptic slices. The results demonstrated that the ultrasound with a frequency of 6.57 MHz could also decrease the firing frequency of pyramidal neurons in human epileptic slices. For the animal study or realistic human therapy, a lower frequency is needed to reduce skull attenuation (Supplementary Figure S11).

Due to the high frequency of 28 MHz, the pulsed ultrasound used in the present study could not generate acoustic cavitation without the microbubbles 72, 73. It is, therefore, likely that the mechanical effect mediated the inhibitory effects of ultrasound on neuronal activities. These results were consistent with previous reports that the electrical activity in the epileptic network was associated with disruption of synaptic inputs, which promoted neuronal hyperexcitability and hyper-synchronization through an increase in excitatory neurotransmission as well as a decrease of inhibitory neurotransmission or GABA, leading to neuronal hyper-excitability 56, 74. Because of differences in the acoustic modal, frequency, acoustic intensity etc. used in the primate experiments as well as human epileptic slice, the inhibitory mechanism of ultrasound might be entirely different. Further studies are needed to understand the different effects.

## Conclusions

Herein, we demonstrated, for the first time, that low-intensity pulsed ultrasound could improve behavioral outcomes in the non-human primate models of epilepsy and suppress abnormal epileptiform activities on slices harvested from epileptic patients. In the non-human primate model of epilepsy, 30 minutes of ultrasound treatment significantly reduced total seizure counts for 8 hours and seizure frequency per hour. In human epileptic slices, ultrasound stimulation could inhibit epileptiform activities with an efficiency exceeding 65%, probably due to the adjustment of synaptic inputs by the increased neuronal excitability of local inhibitory neurons. Our study suggests that low-intensity pulsed ultrasound could suppress epileptiform activities and might provide a potential clinical treatment for epilepsy.

## Supplementary Material

Supplementary figures and tables.
