# Supplementary material for: Non-invasive ultrasonic neuromodulation of neuronal excitability for treatment of epilepsy
Source: Theranostics. 2020 Apr 12;10(12):5514–26. doi: 10.7150/thno.40520 (PMC7196311; doi:10.7150/thno.40520)

Figure S1: Construction and characterization of ultrasound waveforms with fundamental frequency of 750 kHz, acoustic pressure of 0.35 MPa (I_{SPPA}= 2.02 W/cm^2), TBD of 300 μs, PRF of 1000 Hz, SD of 200 ms, and ISI of 5 seconds for stimulation of monkey models. (A) A cross-sectional diagram of ultrasound energy delivered to epileptogenic foci of monkey. (B) Acoustic field in XZ plane measured by a needle hydrophone (Precision Acoustics, Dorchester, Dorset, UK). (C) Ultrasound pressure mapping acquired by a commercial finite element method (FEM) software COMSOL Multiphysics. Acoustic field measured in XY plane by the OptiSon® Ultrasound Beam Analyzer (Onda, USA), White scale bar = 1 cm.

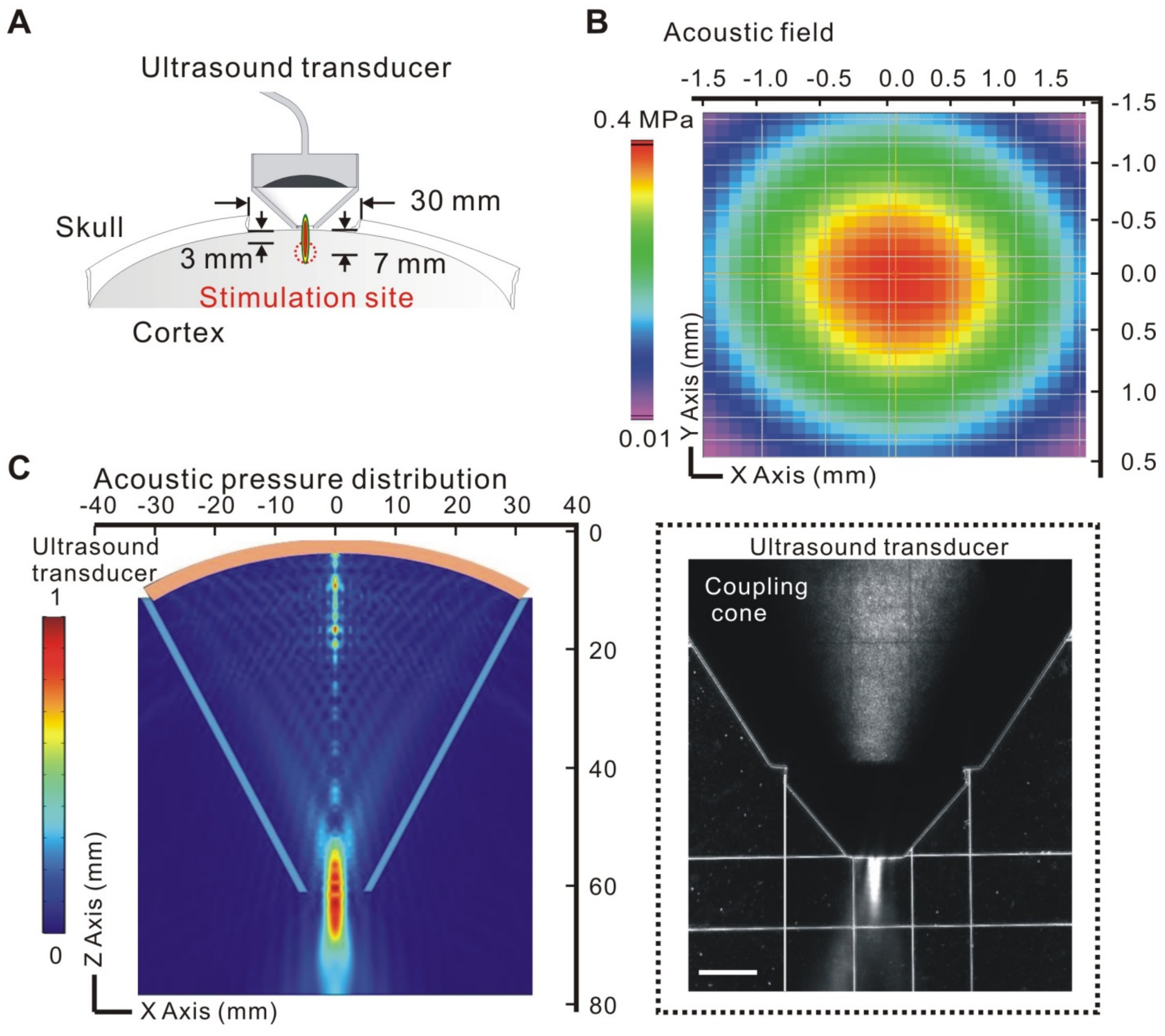

Figure S2: Safety evaluation of 30 min ultrasound stimulation on epileptic monkey model. (A) Temperature monitoring after 30 min ultrasound stimulation was relatively small (0.69 ± 0.044°C) (B) An example of histological examination indicated that ultrasound did not cause pathological damage in the epileptic monkey model.

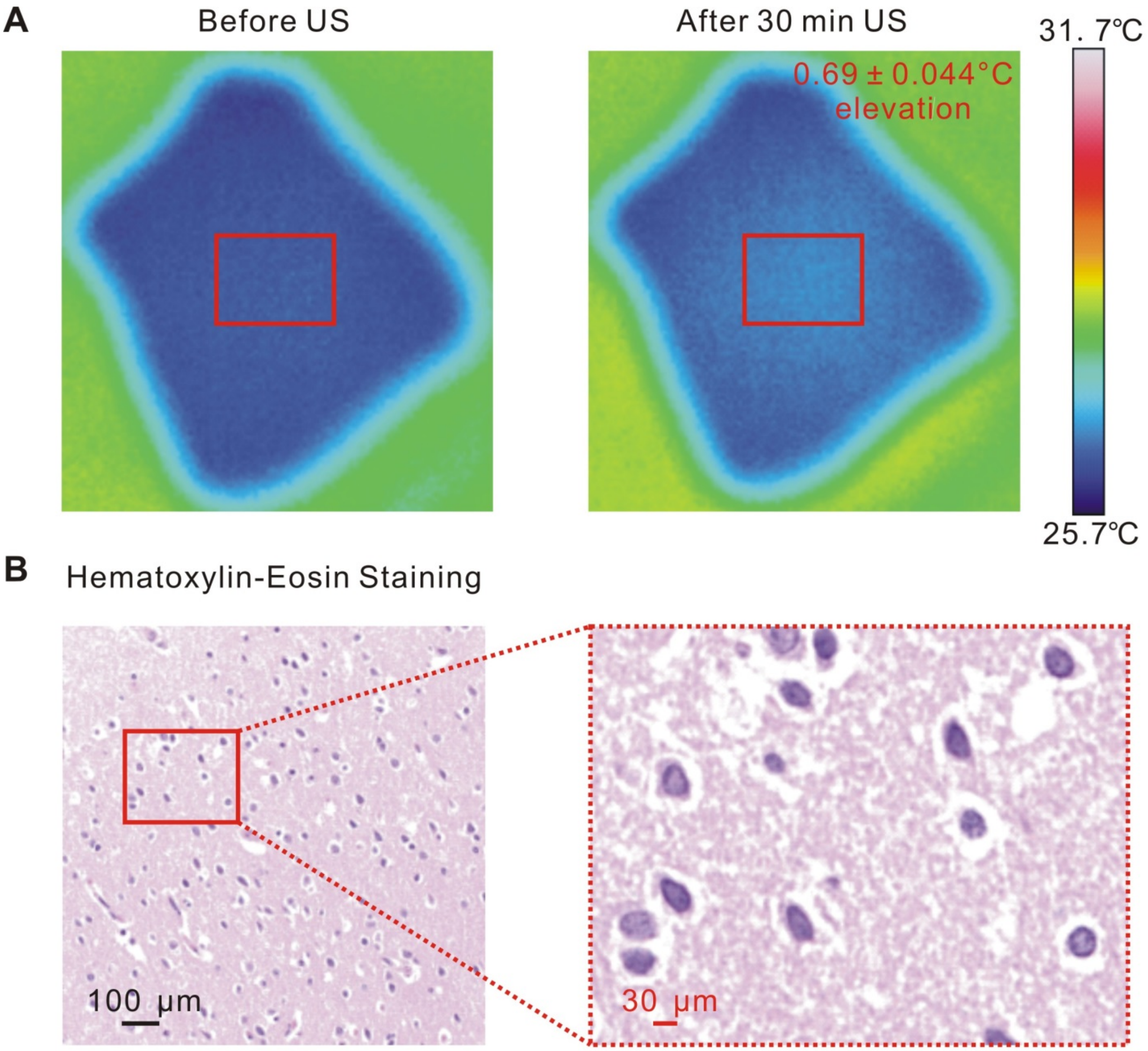

Figure S3: Evaluation of any audible sound produced by ultrasound transducer on epileptiform activities. (A) A cross-sectional diagram of ultrasound transducer was placed outside away from the monkey brain. (B) An example of epileptiform activities and calculated data indicated that ultrasound transducer placed away from the monkey brain had no significant effect on the epileptiform activities of epileptic monkey.

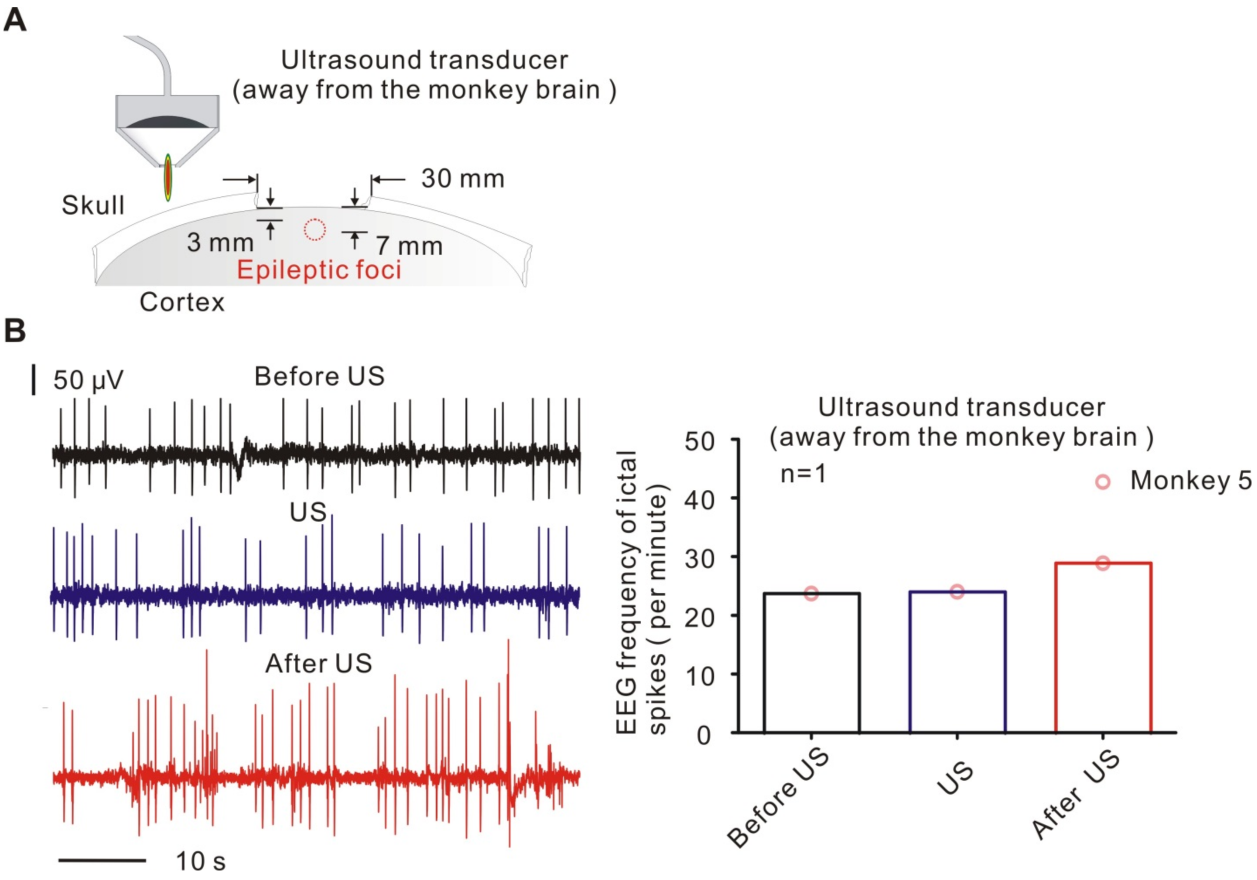

Table S1: Clinical characteristics of 12 TLE patients and 4 high-grade glioma patients.

**No.**	**Age (y)**	**Sex**	**Side**	**EEG**		**MRI***	**Seizures**				**Neuropathology**		
				IEDs	Ictal EEG		Auras	Automati-sm	unilateral TCS	GTCs	FCD	MTS	others
**Twelve TLE patients**													
1	2	M	L	+		+			+	+	+	+	
2	25	F	R	+	+		+	+	+		+		
3	43	M	R	+		+	+		+		+		GNT
4	31	M	R	+	+	+		+	+	+	+	+	
5	31	F	L	+		+	+	+					DNET
6	18	F	L	+		+		+	+	+	+	+	
7	12	M	L	+		+		+	+		+		
8	19	M	L	+	+	+	+	+	+		+	+	
9	18	F	L	+	+	+	+	+	+		+	+	
10	10	F	L	+	+	+	+	+	+	+	+		
11	32	M	L	+		+	+	+	+	+		+	CHM
12	2	F	R	+		+	+	+	+	+			
13	24	M	R	+	+	+	+	+		+			
14	1	F	R	+		+	+	+	+	+	+	+	
15	57	F	L	+	+	+	+		+	+	+		
**Four high-grade glioma patients**													
1	51	F	R			+							GBM
2	41	F	L			+							AA
3	53	M	R			+							GBM
4	47	M	R			+							DA

Note. +, the patient had the signs and symptoms listed in this table; *, MRI signal abnormalities; GTCs, generalized tonic-clonic seizures; FCD, focal cortical dysplasia; MTS, mesial temporal sclerosis; IEDs, interictal epileptiform discharges; TCS, tonic-clonic seizures; GNT, glioneuronal tumor; DNET, dysembryoplastic neuroepithelial tumor; CHM, cavernous hemangioma; GBM, glioblastoma multiforme; AA, anaplastic astrocytoma; DA, diffuse astrocytoma.

Figure S4: MRI imaging, EEGs recording and pathological assessments in a TLE patient. (A) Coronal turbo SE T2-weighted image (left) and transverse T2-weighted FLAIR image (right) showing the blurring of the gray matter-white matter boundaries and increased signal intensity in the temporal lobe (red arrow). (B) Epileptiform discharges in the TLE patient observed in interictal EEG. (C) Pathological sections from biopsy specimen of a TLE patients showing the key features of the biopsy specimen from TLE patient, including focal cortical dysplasia**,** and change of cellular morphology (Black bar, 1 mm; Red bar, 100 μm; Blue bar, 20 μm). (D) HE staining indicates an example of neuronophagia in which a dying neuron is surrounded by microglial cells (Black bar, 100 μm; Red bar, 30 μm). (E) Bielschowsky sliver staining indicates an example of microvacuoles in neurons (Black bar, 100 μm; Red bar, 30 μm).

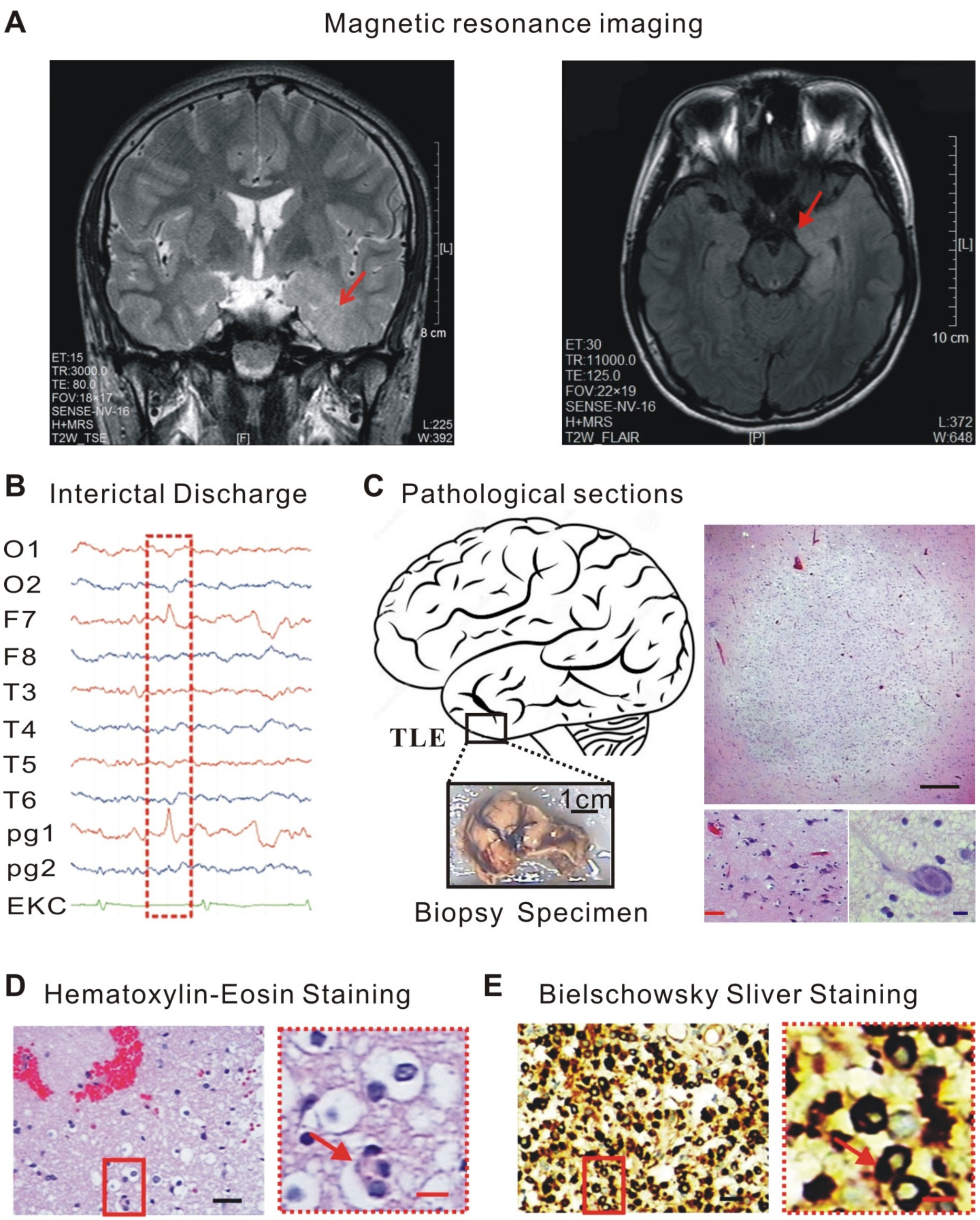
A
Magnetic resonance imaging
ET:15
TR:3000.0
TE: 80.0
FOV:18×17
SENSE-NV-16
H+MRS
T2W_TSE
[F]
[L]
8 cm
L:225
W:392
ET:30
TR:11000.0
TE:125.0
FOV:22×19
SENSE-NV-16
H+MRS
T2W_FLAIR
[P]
[L]
10 cm
L:372
W:648
B
Interictal Discharge
O1
O2
F7
F8
T3
T4
T5
T6
pg1
pg2
EKC
C
Pathological sections
TLE
1cm
Biopsy Specimen
D
Hematoxylin-Eosin Staining
E
Bielschowsky Sliver Staining

Figure S5: Construction and characterization of miniaturized ultrasound stimulation systems used for stimulation of brain slices. (A) Schematic illustration of the experimental setups for transmitting pulsed ultrasound waveforms into brain slices. Brain slices were visually recorded in the recording chamber which was perfused with artificial cerebrospinal fluid (ACSF). Pulsed surface acoustic waves (SAW) were generated by an arbitrary waveform generator and amplified by a power amplifier. IDTs, interdigital transducers. (B) Cross-sectional diagram of the ultrasound neuro-modulation chip was compatible with the recording chamber for patch-clamp recording. The distribution of the acoustic field was monitored in the recording chamber by a Laser Doppler Velocimetry. The energy of ultrasound was localized to the substrate surface, facilitating the stimulation of region-specific slice with relatively small input power. (C) Illustration of sonication pulsing schemes. TBD indicates tone-burst-duration; PRF, pulse repetition frequency.

50

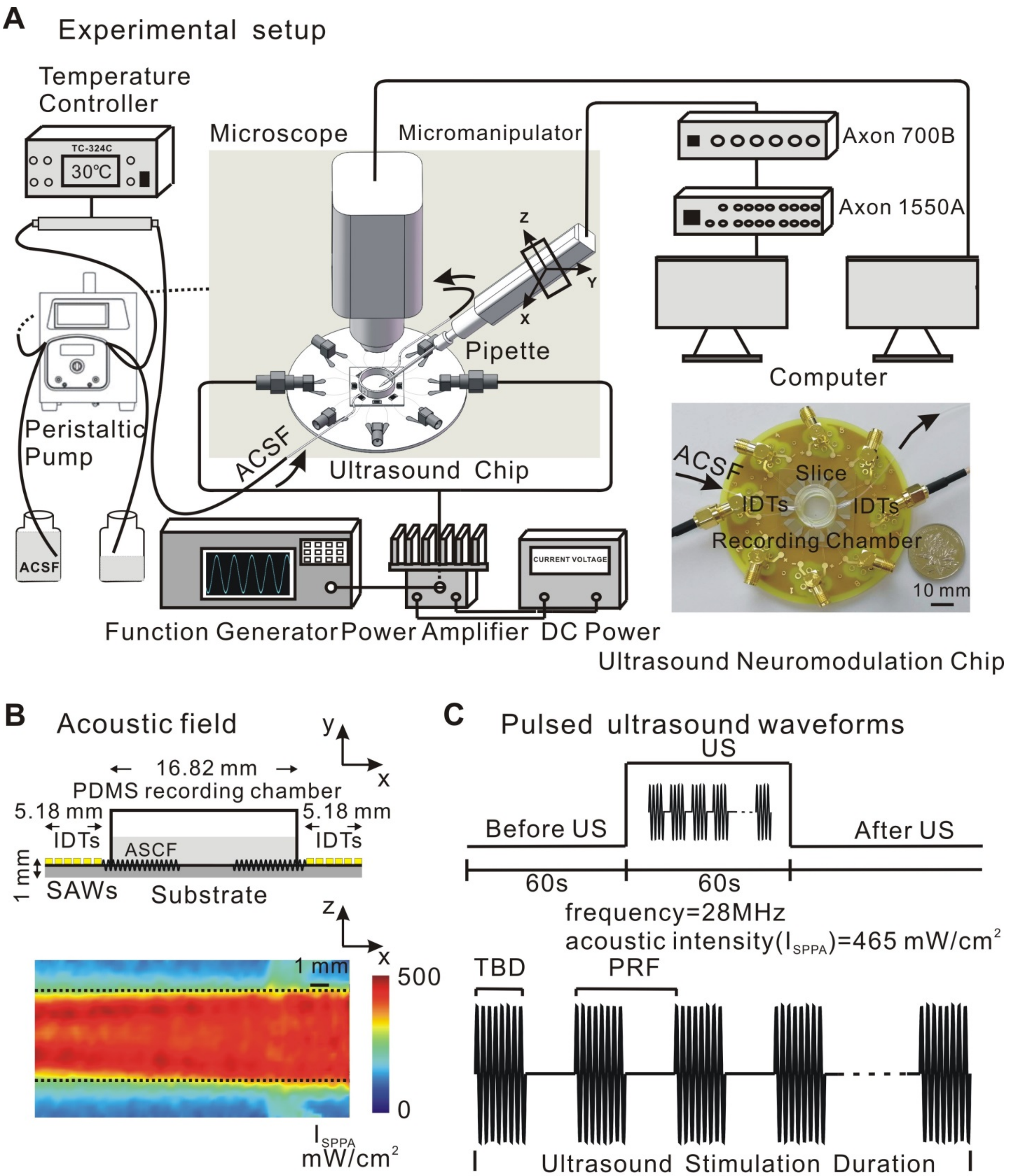
A
Experimental setup
Temperature Controller
TC-324C
30℃
Microscope
Micromanipulator
Axon 700B
Axon 1550A
Pipette
Computer
Peristaltic Pump
ACSF
Ultrasound Chip
ACSF
Slice
IDTs
IDTs
Recording Chamber
10 mm
ACSF
CURRENT VOLTAGE
Function Generator
Power Amplifier
DC Power
Ultrasound Neuromodulation Chip
B
Acoustic field
16.82 mm
PDMS recording chamber
5.18 mm
5.18 mm
IDTs
IDTs
ASCF
1 mm
SAWs
Substrate
1 mm
500
0
I SPPA mW/cm2
C
Pulsed ultrasound waveforms
US
Before US
After US
60s
60s
frequency=28MHz
acoustic intensity(I SPPA)=465 mW/cm2
TBD
PRF
Ultrasound Stimulation Duration

Figure S6: Bi-modal effect of ultrasound stimulation with 60 seconds pulsed ultrasound waveforms with fundamental frequency of 28 MHz**,** and acoustic pressure of 0.13 MPa (I_{SPPA}= 465 mW/cm^2) on neuronal excitability in mice cortical slices. (A) Seven neurons recorded from cortical slices of mice showing an effective increase in the frequency of action potentials induced by 60 seconds ultrasound stimulation with 0.5 ms ultrasound pulses containing 14000 acoustic cycles at pulse repetition frequency (PRF) of 1000 Hz. (B) Eight neurons recorded from cortical slices of mice showing an effective decrease in the frequency of action potentials induced by 60 seconds of ultrasound stimulation with 5 ms ultrasound pulses containing 140000 acoustic cycles at pulse repetition frequency (PRF) of 100 Hz.

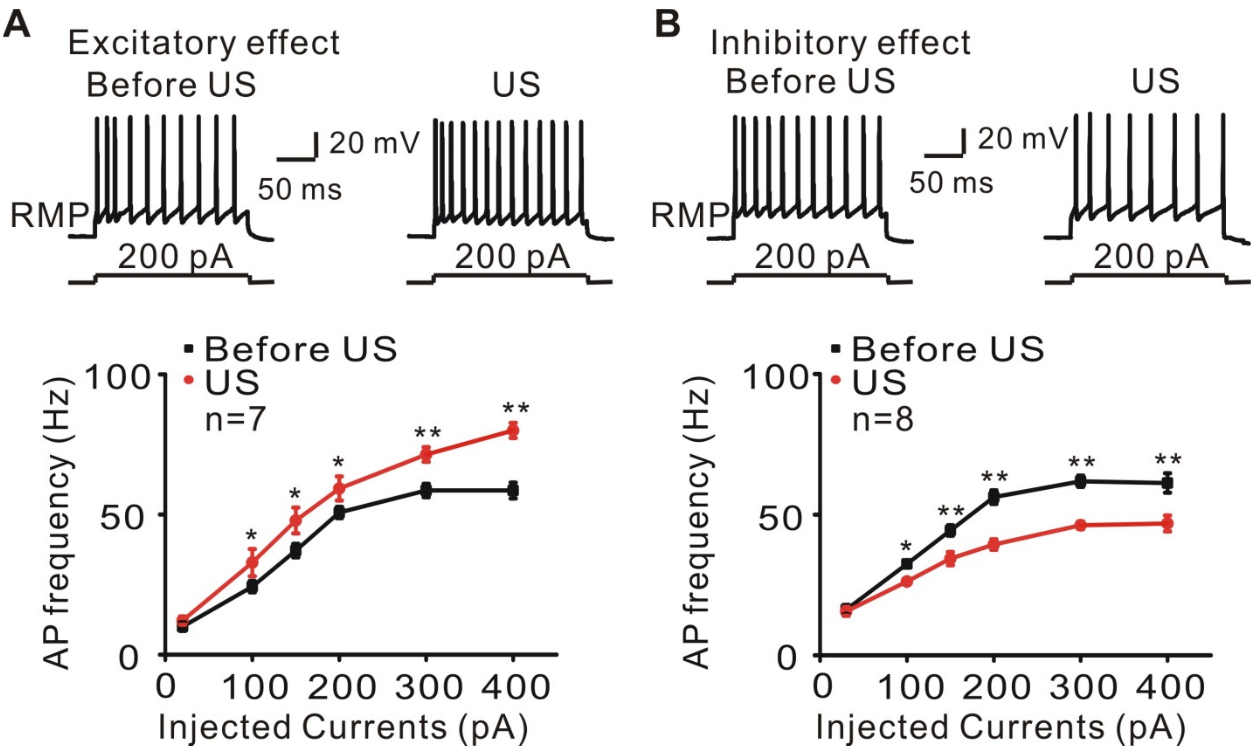

Figure S7: Ultrasound stimulation has no effect on properties of EPSCs and IPSCs. (A) No significant change was observed before US and during US in the amplitude, 10% - 90% rise-time, and half-width of EPSCs from 12 neurons (amplitude: before US: 99.48 ± 8.96 pA; US: 100.22 ± 9.18 pA; rise-time: before US: 1.83 ± 0.04 ms; US: 1.84 ± 0.04 ms; half-width: before US: 5.99 ± 0.09 ms; US: 5.97 ± 0.09 ms). (B) No significant change was observed before US and during US in the amplitude, 10% - 90% rise-time, and half-width of IPSCs from 13 neurons (amplitude: before US: 91.34 ± 9.53 pA; US: 92.98 ± 10.18 pA; rise-time: before US: 4.89 ± 0.05 ms; US: 4.86 ± 0.07 ms; half-width: before US: 17.14 ± 0.21 ms; US: 17.13 ± 0.18 ms).

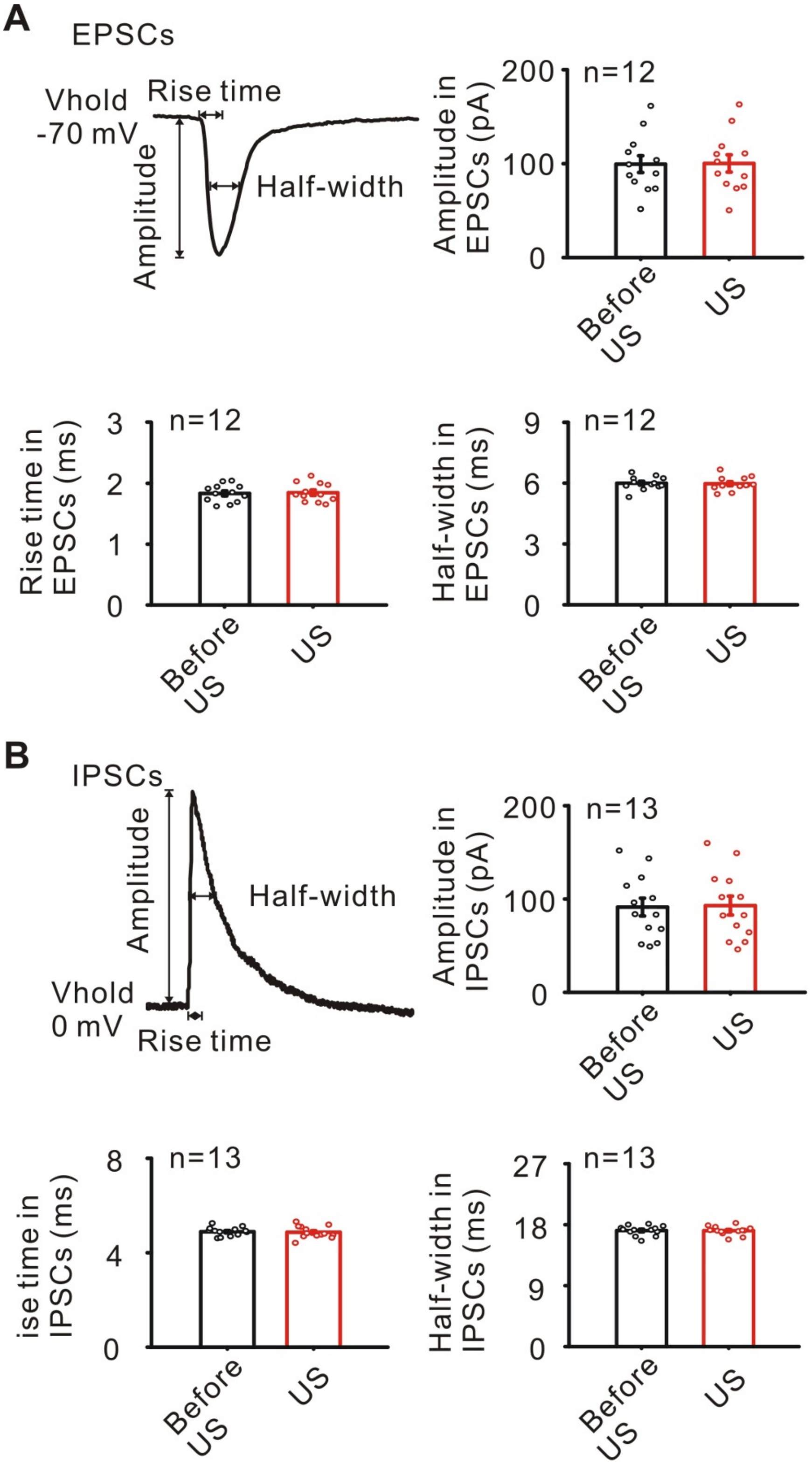
A
EPSCs
Vhold
-70 mV
Rise time
Half-width
Amplitude
n=12
Amplitude in EPSCs (pA)
Rise time in EPSCs (ms)
Half-width in EPSCs (ms)
Before US
US
B
IPSCs
Vhold
0 mV
n=13
Amplitude in IPSCs (pA)
ise time in IPSCs (ms)
Half-width in IPSCs (ms)

Figure S8: Inhibitory synaptic receptor antagonist, SR95531, blocks the inhibitory effect of epileptiform activities induced by ultrasound stimulation. (A) Representative traces of epileptiform discharges recorded from TLE patient's slices pretreated with SR-95531 (middle) in different recording phases (upper, 30s Before US, 60s US and After US). The events of epileptiform discharges did not change by ultrasound stimulation. (B-C) After adding GABA receptor antagonist, ultrasound stimulation hardly changed the normalized frequency of epileptiform discharges in 8 neurons (US: 1.02 ± 0.027 Hz; After US: 1.04 ± 0.026 Hz; P = 0.29, one-way ANOVA followed by LSD). (D-E) Ultrasound stimulation also did not change the waveform of spikes (326.18 ± 46.43 pA to 328.54 ± 41.62 pA, P = 0.31, Student's paired t-test for mean spikes amplitude; 0.525 ± 0.03 ms to 0.515 ± 0.05ms, P = 0.39, Student's paired t-test for mean spikes half-width).

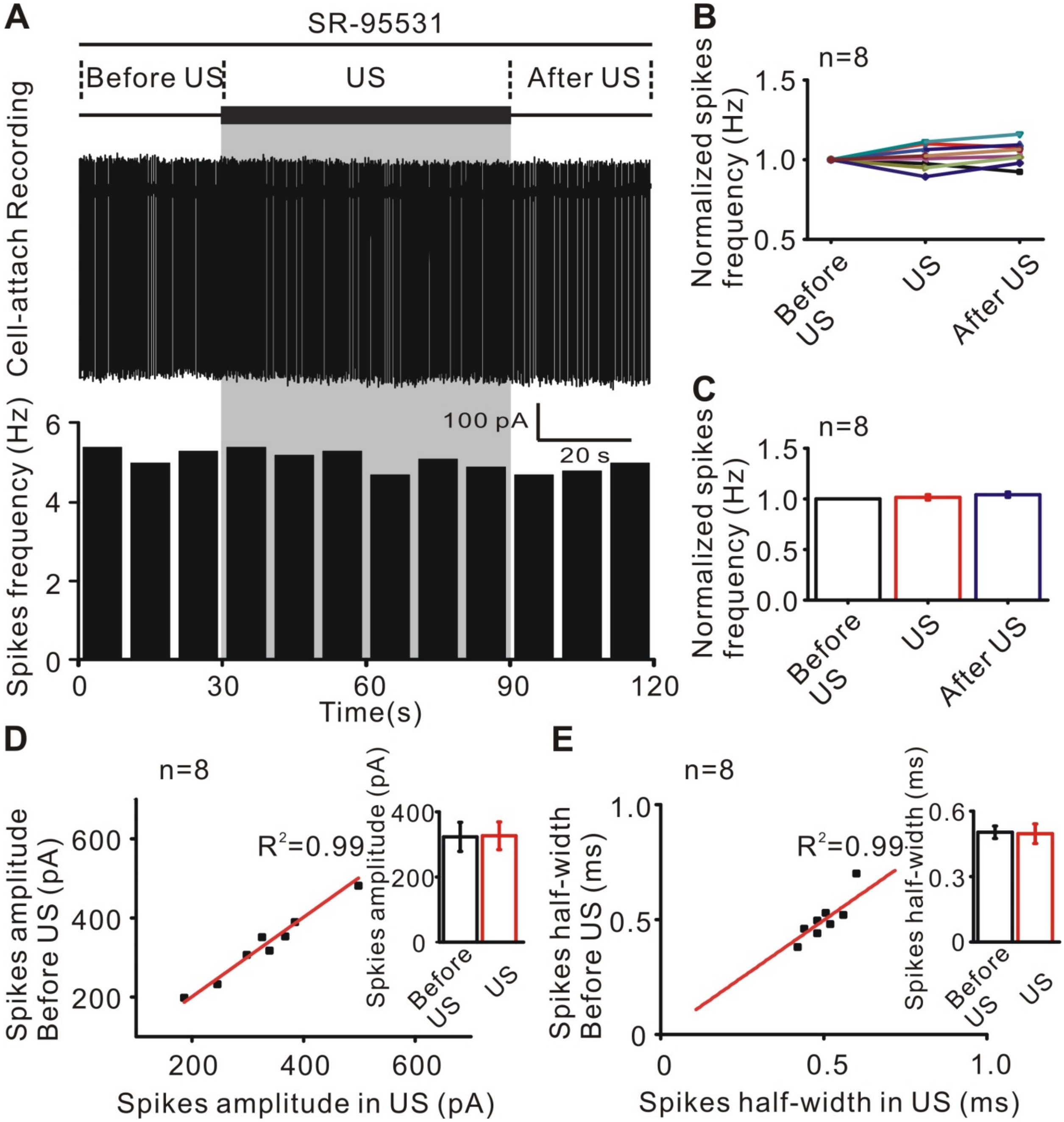
A
SR-95531
Before US
US
After US
Cell-attach Recording
100 pA
20 s
Spikes frequency (Hz)
0
2
4
6
0
30
60
90
120
Time(s)
B
n=8
Normalized spikes frequency (Hz)
0.5
1.0
1.5
Before US
US
After US
C
n=8
Normalized spikes frequency (Hz)
0.0
0.5
1.0
1.5
Before US
US
After US
D
n=8
Spikes amplitude Before US (pA)
200
400
600
R^2=0.99
Spikes amplitude in US (pA)
Spkies amplitude (pA)
0
200
400
Before US
US
E
n=8
Spikes half-width Before US (ms)
0
0.5
1.0
R^2=0.99
Spikes half-width in US (ms)
Spikes half-width (ms)
0
0.3
0.6
Before US
US

Figure S9: Monitoring of temperature during ultrasound stimulation. (A) Temperature elevation was measured during ultrasound stimulation using a thermal infrared imager. The red rectangle indicates the electrophysiological recorded area and the range of temperature monitoring. IDTs, interdigital transducers. (B) A relatively small temperature elevation was observed during 60 second ultrasound stimulation and there was no significant difference between two parameters (less than 0.64 ± 0.036°C and 0.63 ± 0.007°C). (C-D) The temperature profiles over the entire intervention period were visualized for different ultrasonic parameters.

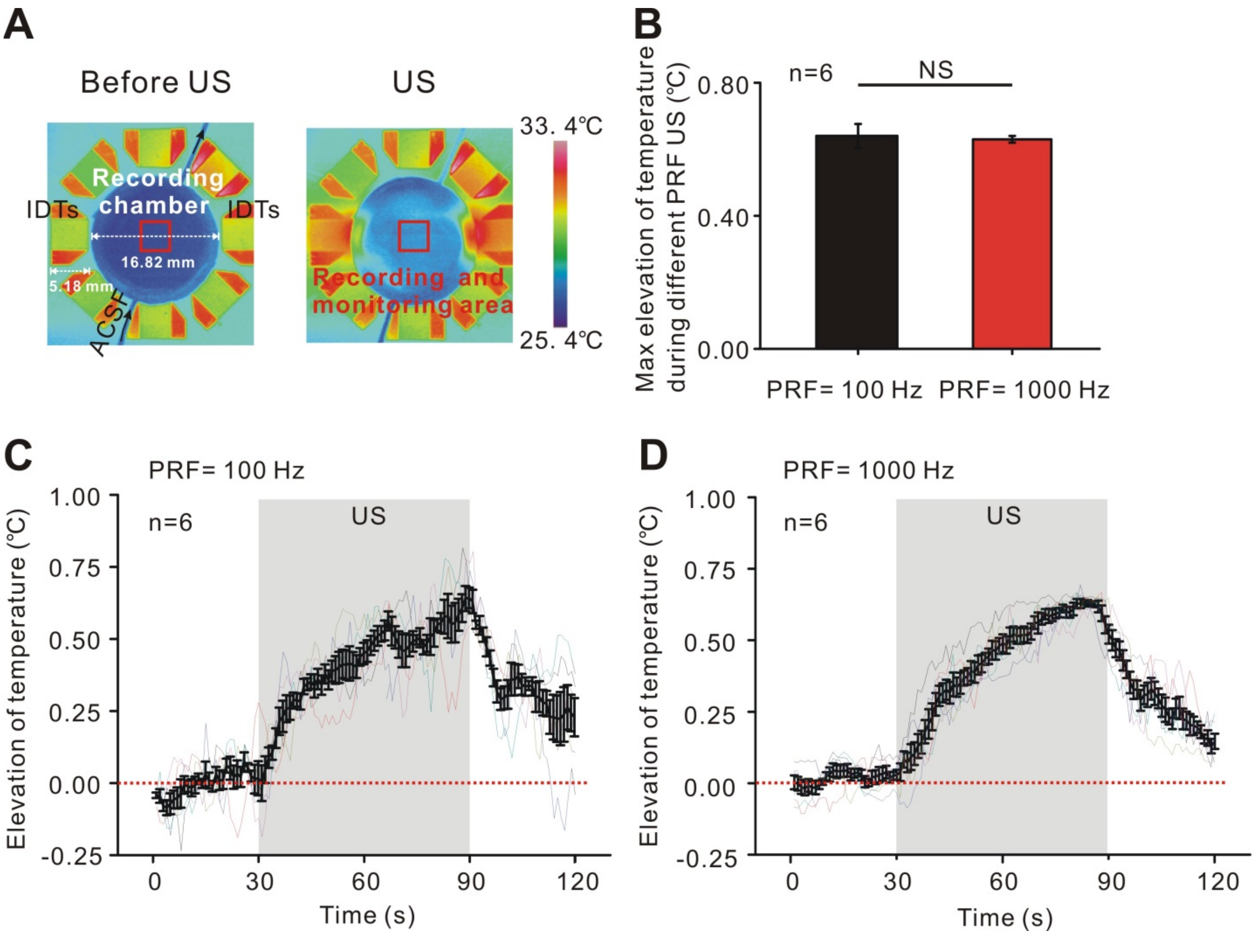

Fig. S10: Evaluation of temperature elevation produced by ultrasound transducer on epileptiform activities. The temperature elevation in ACSF perfusion (2°C) had no effect on the epileptiform discharge (A-C) and neuronal excitabilities (D-E) in epileptic brain slices.

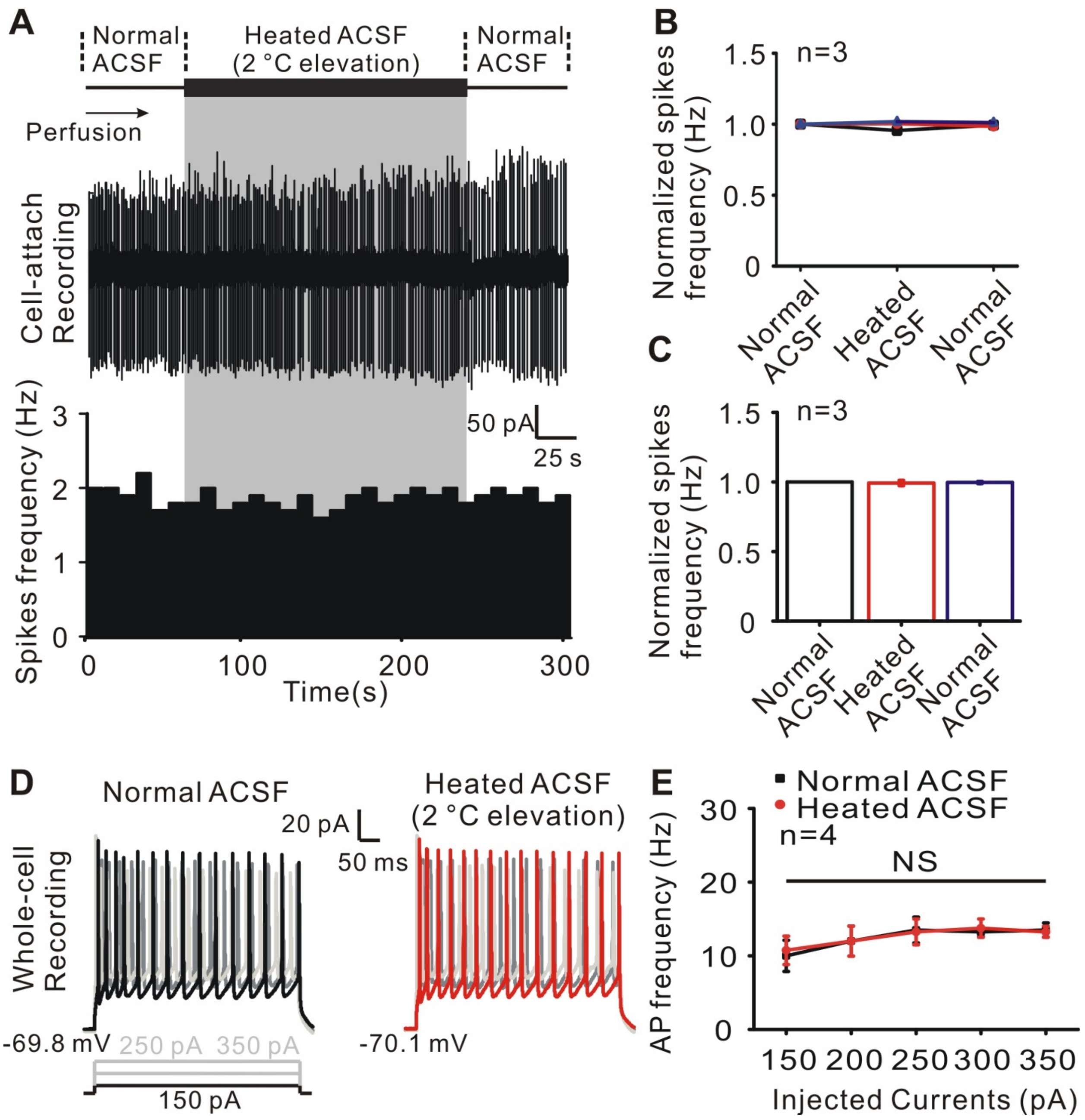

Fig. S11: Evaluation of acoustic resonant frequency of ultrasound neuro-modulation chip on epileptiform activities. Ultrasound at 6.57 MHz frequency could also decrease the firing frequency of pyramidal neurons in human epileptic slices (A-B).

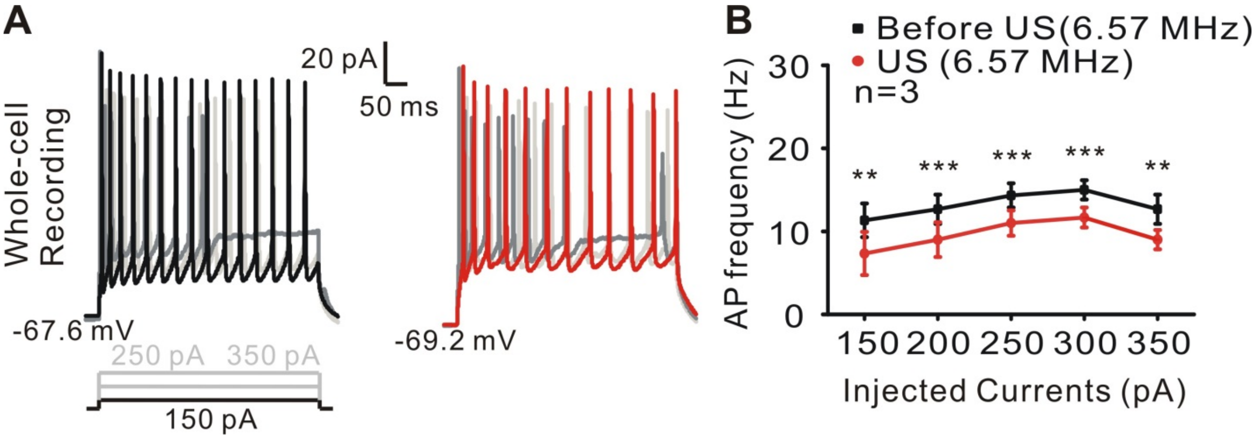

Supplement: Supplementary file 1 — Supplementary figures and tables. [file thnov10p5514s1.pdf]
